# Machine Learning to Quantify Physical Activity in Children with Cerebral Palsy: Comparison of Group, Group-Personalized, and Fully-Personalized Activity Classification Models

**DOI:** 10.3390/s20143976

**Published:** 2020-07-17

**Authors:** Matthew N. Ahmadi, Margaret E. O’Neil, Emmah Baque, Roslyn N. Boyd, Stewart G. Trost

**Affiliations:** 1 Institute of Health and Biomedical Innovation at Queensland Centre for Children’s Health Research, Queensland University of Technology, South Brisbane 4101, Australia; matthewnguyen.ahmadi@hdr.qut.edu.au (M.N.A.); e.baque@griffith.edu.au (E.B.); 2 Faculty of Health, School of Exercise and Nutrition Sciences, Queensland University of Technology, Kelvin Grove 4059, Australia; 3 Department of Rehabilitation and Regenerative Medicine, Columbia University Irving Medical Center, New York, NY 10032, USA; mo2675@cumc.columbia.edu; 4 School of Allied Health Sciences, Griffith University, Gold Coast 4215, Queensland, Australia; 5 Queensland Cerebral Palsy and Rehabilitation Research Centre, UQ Child Health Research Centre, Faculty of Medicine, The University of Queensland, South Brisbane 4101, Australia; r.boyd@uq.edu.au

**Keywords:** accelerometers, wearable sensors, exercise, measurement, GMFCS level

## Abstract

Pattern recognition methodologies, such as those utilizing machine learning (ML) approaches, have the potential to improve the accuracy and versatility of accelerometer-based assessments of physical activity (PA). Children with cerebral palsy (CP) exhibit significant heterogeneity in relation to impairment and activity limitations; however, studies conducted to date have implemented “one-size fits all” group (G) models. Group-personalized (GP) models specific to the Gross Motor Function Classification (GMFCS) level and fully-personalized (FP) models trained on individual data may provide more accurate assessments of PA; however, these approaches have not been investigated in children with CP. In this study, 38 children classified at GMFCS I to III completed laboratory trials and a simulated free-living protocol while wearing an ActiGraph GT3X+ on the wrist, hip, and ankle. Activities were classified as sedentary, standing utilitarian movements, or walking. In the cross-validation, FP random forest classifiers (99.0–99.3%) exhibited a significantly higher accuracy than G (80.9–94.7%) and GP classifiers (78.7–94.1%), with the largest differential observed in children at GMFCS III. When evaluated under free-living conditions, all model types exhibited significant declines in accuracy, with FP models outperforming G and GP models in GMFCS levels I and II, but not III. Future studies should evaluate the comparative accuracy of personalized models trained on free-living accelerometer data.

## 1. Introduction

Cerebral palsy (CP) is the most common physical disability in childhood, with a prevalence of 2.1 cases per 1000 live births [1,2]. Specifically, among children with CP, physical activity levels decrease by 34% to 47% as children progress from early childhood through adolescence and such children accumulate less physical activity than their typically developing peers [3,4,5]. Low physical activity levels, in addition to associated neuromotor and functional limitations, impact the long-term health and well-being of children with CP [6,7,8]. In light of this evidence, researchers and clinicians have focused on delivering interventions to increase habitual physical activity and decrease sedentary behavior [9,10,11]. Historically, the effectiveness of these interventions has been evaluated using self-reports of physical activity. Although self-reports are low-cost and easy for participants to complete, they are subject to considerable social desirability and recall bias, and therefore may not be sufficiently valid or reliable for an assessment of clinically important changes in physical activity [12]. As a result, an increasing number of studies involving children with CP are employing device-based measures of physical activity and sedentary behavior [13,14].

Accelerometer-based motion sensors have become the method of choice for assessing physical activity in children [15,16]. However, the atypical gait patterns and lower mechanical efficiency of children with CP mandate the development of bespoke algorithms for reducing the accelerometer output for physical activity metrics [12]. A number of studies have derived intensity cut-points to categorize accelerometer data as sedentary (SED), light (LPA), or moderate-to-vigorous physical activity (MVPA) among children with CP [12,17,18,19,20]. This research has enabled researchers and clinicians to quantify the physical activity levels of children with CP and examine compliance with physical activity recommendations. However, validation studies involving independent samples of children with CP have demonstrated that cut-point approaches misclassify MVPA as LPA or SED activity 30% of the time and dramatically underestimate the physical activity levels of children with more severe motor impairments [21]. As such, there is a critical need to investigate new accelerometer data processing methods that potentially provide more accurate assessments of physical activity and sedentary behavior in children with CP.

Pattern recognition methodologies, such as those utilizing machine learning approaches, have the potential to significantly improve the accuracy of accelerometer-based assessments of physical activity among children with CP. In a previous study [22], we developed machine learning activity classification models for ambulatory children with CP for accelerometers worn on the wrist, hip, and a combination of the wrist and hip [22]. The resultant Random Forest and Support Vector Machine physical activity classification models classified sedentary activities with 96% to 98% accuracy, standing utilitarian movements (e.g., folding laundry) with 83% to 97% accuracy, and walking with 90% to 97% accuracy. However, the study sample was relatively small and predominantly comprised of children functioning at Gross Motor Function Classification System (GMFCS) levels I and II. Consequently, the study could not examine the classification accuracy in children with more severe movement impairments who ambulate with the assistance of crutches and walkers. Furthermore, prior work in typically developing children has demonstrated that other accelerometer placements, such as the ankle or a combination of the ankle and wrist, provide a higher classification accuracy [23,24]. However, the utility of activity classification models trained on accelerometer data from the ankle has not been examined in children with CP.

Typically, machine learning classification models are trained using accelerometer data from groups of participants and applied to new samples assuming that the new participants have the same movement patterns as those in the training sample. This presupposition may not be valid in the CP population, given the significant heterogeneity in relation to movement impairment and functional capacity. An alternative approach, which has not been investigated in children with CP, is to train and deploy group-personalized models based on the GMFCS level. In this approach, classification models are trained on accelerometer data from a specific GMFCS group and applied to children of the same GMFCS classification. It has previously been demonstrated that GMFCS-specific decision tree models for classifying the physical activity intensity in children with CP outperform “one-size-fits-all” models by accounting for differences in the energy cost of locomotion [21].

Although group-personalized models may account for broadly defined differences in functional ability, fully-personalized or individually-calibrated models may provide even more accurate physical activity predictions by accounting for finer grained differences in functional ability related to the child’s age, height, motor distribution, and movement disorder. In a study of healthy adults, personalized activity classification models, trained on less than five minutes of accelerometer data, exhibited a significantly greater classification accuracy than group models [25]. On average, the overall classification accuracy increased by 26 percentage points from 71% to 97%, while walking recognition improved from 65% to 99%. More recently, Carcreff et al. [26] reported significantly improved walking bout detection and walking speed estimation in children with CP after applying personalized thresholds for the left and right mid-swing shank angular velocity. To the best of our knowledge, the relative accuracy and utility of fully-personalized machine learning activity classification models have not been examined in children with CP.

To date, most activity classification algorithms have been developed using data collected in controlled laboratory environments, which may not be generalizable to free-living settings [27,28,29]. Developing and validating classification models in the laboratory may be unrealistic because only a limited number of activities are included and fixed-duration activity trials without natural transitions between activities fail to replicate the episodic nature of movement behaviors displayed in true free-living environments [28]. If accelerometer-based physical activity classification models for children with CP are to be used in field-based studies, it is important to evaluate their accuracy under scenarios that replicate free-living environments.

To address these gaps in the research literature, the purpose of the current study was to evaluate and compare the accuracy of group, group-personalized, and fully-personalized machine learning physical activity classification models in children with CP. To examine the effects of accelerometer placement, models were trained and tested using accelerometer data from the hip, wrist, and ankle, and all two- and three-placement combinations. To assess the validity under conditions that more closely replicated intervention studies conducted in real-world settings, the classification accuracy was evaluated and compared in a simulated free-living trial.

## 2. Materials and Methods

### 2.1. Participants

A total of 38 ambulatory youth with CP participated in the study. Participants were recruited from two sites—St Christopher’s Hospital for Children, Philadelphia, USA and the Queensland Centre for Children’s Health Research, Brisbane, Australia. The inclusion criteria were as follows: Diagnosis of CP at Gross Motor Function Classification System (GMFCS) level I, II, or III; between the ages of 6 and 18 years; and able to follow verbal instructions. Parents and/or health care providers (doctors or therapists) verified that participants were able to complete the study protocols. Participants were excluded from the study if they had undergone orthopedic surgery within the last 6 months, received lower extremity Botulinum toxin A injections within the last 3 months, or experienced a recent musculoskeletal injury or had a medical condition limiting their ability to complete the physical activity protocol. The descriptive characteristics are displayed in Table 1. The study was approved by each university’s Institutional Review Board. Prior to participation, parents provided written consent and children written assent.

### 2.2. Individual Activity Trials

Participants were randomized to complete one of two activity protocols. The protocol was completed in a single two-hour session and consisted of five activity trials. Briefly, the activity trials included in protocol one were as follows: (1) Supine rest (lying down and resting, but not sleeping); (2) sitting while continuously writing or coloring in a picture; (3) active video game (playing an interactive video game designed specifically for children with CP); (4) comfortable-paced walk in a 25 m course (walking at a pace with the instructions “walk like when you are at the mall or walking in your neighborhood or at school, but you are not in a hurry.”); and (5) brisk-paced walk in a 25 m course (walking at a pace with the instructions “walk at a fast pace like when you are hurrying to get to class after the bell has rung or you are hurrying to cross the street”). In protocol two, sitting while continuously writing or coloring in a picture was replaced with wiping down a table (standing at a waist-height counter and spraying the counter with water and then wiping the water off). All of the activity trials were 6 min in duration. Activity trials were categorized as one of three activity classes: Sedentary (SED = supine rest and sitting); standing utilitarian movements (SUM = wiping a table and active video game); and walking (WALK = comfortable-paced walk and brisk-paced walk).

### 2.3. Instrumentation

Participants wore an ActiGraph GT3X+ tri-axial accelerometer (ActiGraph Corporation, Pensacola, FL, USA) on the least impaired wrist, hip, and ankle, with the Y-axis pointing vertically. The ActiGraph GT3X+ is a small (4.6 cm × 3.3 cm × 1.5 cm) and lightweight (19 g) monitor that measures acceleration along three orthogonal axes and the sampling frequency for the current study was set to 30 Hz.

### 2.4. Machine Learning Activity Classification Models

Random Forest (RF) classifiers were trained to categorize activities as one of three activity classes using accelerometer data collected at each single placement (wrist, hip, and ankle), and a combination of two placements (wrist and hip = W + H; wrist and ankle = W + A; hip and ankle = H + A) and three placements (wrist and hip and ankle = W + H + A). RF is an ensemble of decision tree models. Each tree is developed based on a bootstrap sample of training data and each node in the tree is split using the best value among a randomly selected sample of features. The decisions from each tree are aggregated and a final model prediction is based on a majority vote [30].

#### 2.4.1. Data Pre-Processing and Feature Extraction

Tri-axial accelerometer data corresponding to the start and end of each activity trial were parsed and annotated with the corresponding activity class label. Features were extracted from 10 s non-overlapping windows. In total, 15 features were extracted from each axis and included the minimum, maximum, mean, variance, standard deviation, skewness, kurtosis, percentiles (25th, 50th, and 75th), zero-crossings, energy, dominant frequency, dominant magnitude, and entropy. Cross-axis correlations and the mean vector magnitude were also extracted. The extracted features have previously been shown to be informative for activity classification in children with CP and typically developing children [22,31].

#### 2.4.2. Model Training and Cross-Validation

Models were trained and cross-validated using the “randomForest” and “caret” packages within R (Version 3.5.3). Prior to model training, minimum Redundancy Maximum Relevance (mRMR) feature selection was used to identify features with a high discriminative ability [32]. Minimum redundancy favors features that have a low dependency on other features, without considering how important they are to the outcome variable, whereas maximum relevance selects features that are the most predictive of the outcome variable. The mRMR selection process is based on a balance between these two algorithms, selecting features that derive a high relevance and low redundancy. Feature selection was constrained to the 10, 15, and 20 best features. The number of trees for each model was kept constant at 500 and the number of features sampled at each tree node in the forest ranged from 3 to 11 features. Group models were trained on data from all 38 participants, while the group-personalized models based on the GMFCS level were only trained on data from participants at the same GMFCS level and fully-personalized models were trained on data from each individual.

For the group and group-personalized models, the out-of-sample classification accuracy was evaluated using leave-one-subject-out cross-validation (LOSO-CV). In LOSO-CV, the model is trained on data from all of the participants except one, which is ‘‘held out’’ and used as the test data set. The process is repeated until all participants have served as the test data, and the performance metrics are aggregated. For the fully-personalized models, the classification accuracy was evaluated using 10-fold cross-validation. With 10-fold cross-validation, the participant’s data are randomly partitioned into 10 subsets and the model is trained on all subsets except one, which is used as the test data set. The process is repeated until all subsets have served as the test data, and the performance metrics are aggregated. The cross-validation classification accuracy was evaluated by computing the overall and class-level accuracy for each GMFCS level.

### 2.5. Simulated Free-Living Evaluation

To evaluate the classification accuracy under conditions that more closely reflected a free-living environment, participants completed a simulated free-living trial in which they performed the following sequence of activities: (1) Sitting on a bench; (2) walking 10 m whilst weaving around cones to a table; (3) standing at the table and completing a puzzle and/or playing with a toy; and (4) walking around the table and back to the bench and sitting. The entire sequence was repeated for 6 min. To obtain ground truth activity class labels, all trials were video recorded with a Go-Pro camera (GoPro, Inc., San Mateo, CA, USA). These video files were subsequently imported into the Noldus Observer XT software (Noldus Information Technology, Wageningen, The Netherlands) for coding of the participant’s activity type as either “SED”, “SUM”, or “WALK”. The Observer software generated a vector of date-time stamps corresponding to the start and finish of each activity, which were used to label the corresponding time segment of the accelerometer data. For each accelerometer placement and model type, the overall and class-level accuracy was calculated for each GMFCS level.

### 2.6. Statistical Evaluation

A 3 × 3 × 7 repeated measures ANOVA was used to examine the effects of the model type, GMFCS level, and placement on the overall accuracy. Significant main effects and interactions were evaluated using tests of simple effects and pre-planned contrasts. Significance was set at an alpha level of 0.05.

## 3. Results

For the group wrist, hip, and ankle classification models, respectively, 15, 15, and 10 features were selected. For the multiple placement W + H, W + A, H + A, and W + H + A classification models, respectively, 15, 10, 10, and 20 features were selected. A complete list of the selected features for each model are reported in Supplemental Document 1.

### 3.1. Leave-One-Subject-Out Cross-Validation

**Figure 1:**
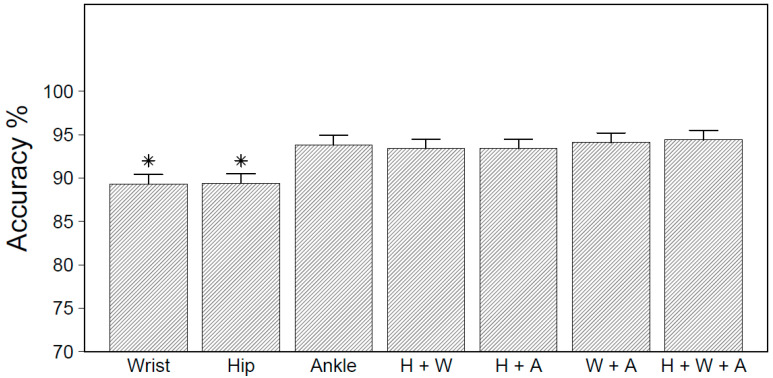
Overall accuracy obtained by placement for leave-one-subject-out cross-validation (LOSO-CV). * Significantly different from ankle and multi-placement models at *p* < 0.05.

There was a significant main effect for placement on the accuracy (F_6,210_ = 11.26, *p* < 0.01), with the wrist and hip exhibiting a significantly lower overall accuracy than all other placements (see Figure 1). Compared to models trained on ankle accelerometer data, there were no significant improvements in the overall accuracy for the two-placement and three-placement models.

**Figure 2:**
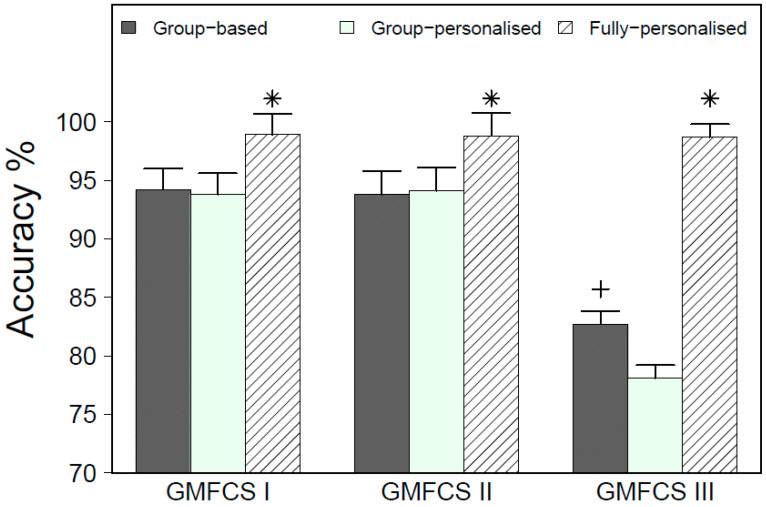
Overall accuracy for model type by GMFCS level for LOSO-CV. * Significantly different from group and group-personalized models; + significantly different from the group-personalized model.

Figure 2 displays the overall accuracy for group, group-personalized and fully-personalized models by GMFCS level, averaged over all placements. There was a significant model type by GMFCS level interaction (F_4,70_ = 33.38, *p* < 0.01), indicating that differences in the classification accuracy varied by GMFCS level. Averaged over all placements, fully-personalized models exhibited a significantly higher accuracy than group and group-personalized models, with the largest differential observed in children at GMFCS III.

Table 2 reports activity class recognition for the group, group-personalized, and fully-personalized models for each accelerometer placement combination by GMFCS level. For all placements and GMFCS levels, the fully-personalized models exhibited > 96.0% recognition accuracy for SED, SUM, and WALK. For the group and group-personalized models, the class level recognition accuracy varied by GMFCS level. Among children at GMFCS I and II, the group and group-personalized models demonstrated excellent classification accuracy for SED (89.1–98.4%) and WALK (88.3–99.6%), and good to excellent classification accuracy for SUM (76.8–93.8%). Among children at GMFCS III, the classification accuracy for the group and group-personalized models for SED (57.7–99.6%), SUM (68.6–92.1%), and WALK (53.5–96.3%) was inconsistent and ranged from poor to excellent, depending on the accelerometer placement configuration. Detailed confusion matrices for each placement, model type, and GMFCS level can be found in Supplemental Document 2.

### 3.2. Simulated Free-Living Trial

**Figure 3:**
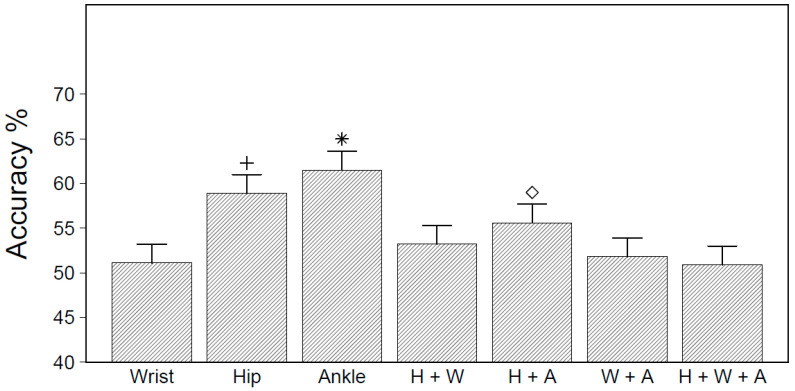
Overall accuracy statistics by accelerometer placement when evaluated under simulated free-living conditions. * Significantly different from all models at *p* < 0.05; + significantly different from all models except H+A; ◇ significantly different from all models except hip.

The overall classification accuracy for the different accelerometer placement configurations under simulated free-living conditions, averaged over all model types and GMFCS levels, is displayed in Figure 3. The ANOVA results indicated a significant main effect for placement (F_6,210_ = 8.90, *p* < 0.01). Tests of simple effects revealed that models trained on accelerometer data from the ankle, hip, or hip and ankle combined had a significantly higher accuracy than all other placement combinations. For all accelerometer placements, however, the overall accuracy statistics during the simulated free-living evaluation were substantially lower compared to the LOSO-CV evaluation and ranged from 50.9% for the H + W + A model to 61.5% for the ankle model.

**Figure 4:**
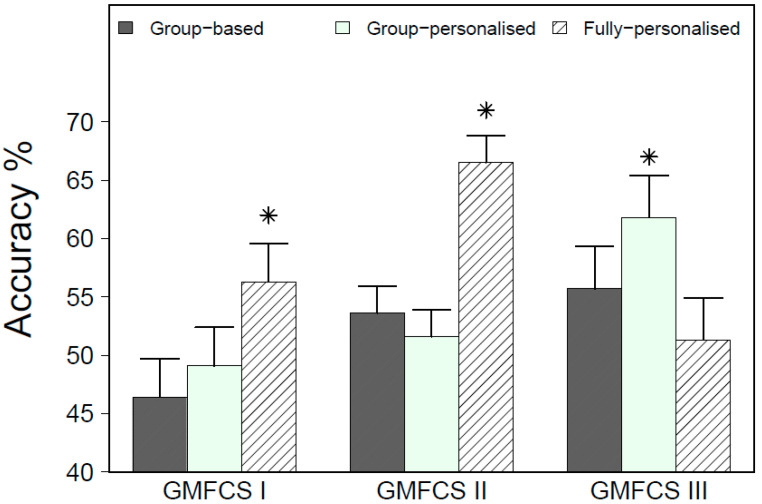
Overall accuracy for group, group-personalized, and fully-personalized Random Forest (RF) classifiers, by GMFCS level, during the simulated free-living evaluation. * Significantly different from other models within GMFCS level at *p* < 0.05.

The simulated free-living accuracy results for the group, group-personalized and fully-personalized models by GMFCS level are displayed in Figure 4. There was a significant model type by GMFCS level interaction (F_4,70_ = 17.94, *p* < 0.01). Among children at GMFCS I and II, fully-personalized models exhibited a significantly higher overall accuracy than the group and group-personalized models. Among children at GMFCS III, however, group-personalized models exhibited a significantly greater overall accuracy than the group and fully-personalized models.

**Figure 5:**
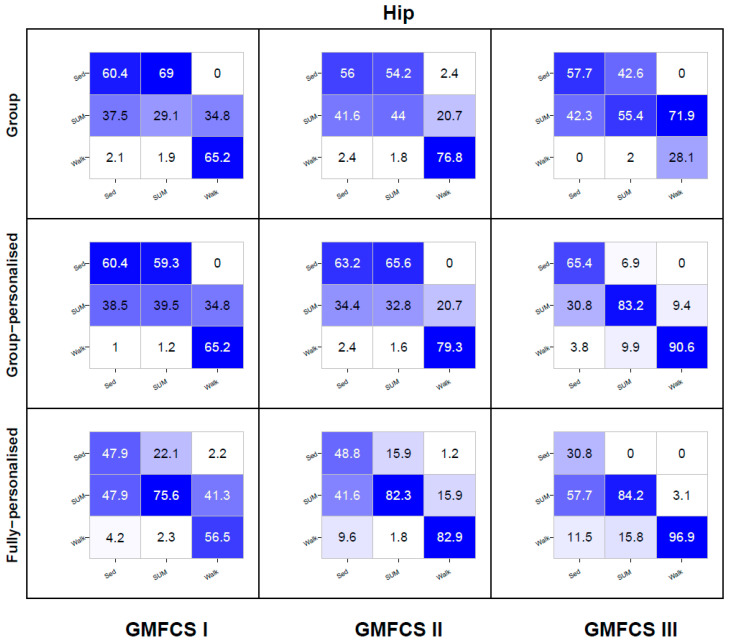
Hip placement activity class recognition for group, group-personalized, and fully-personalized classification models during the simulated free-living trial. Columns represent observed (%); rows represent predictions (%); values on the diagonal represent correct predictions (%).

**Figure 6:**
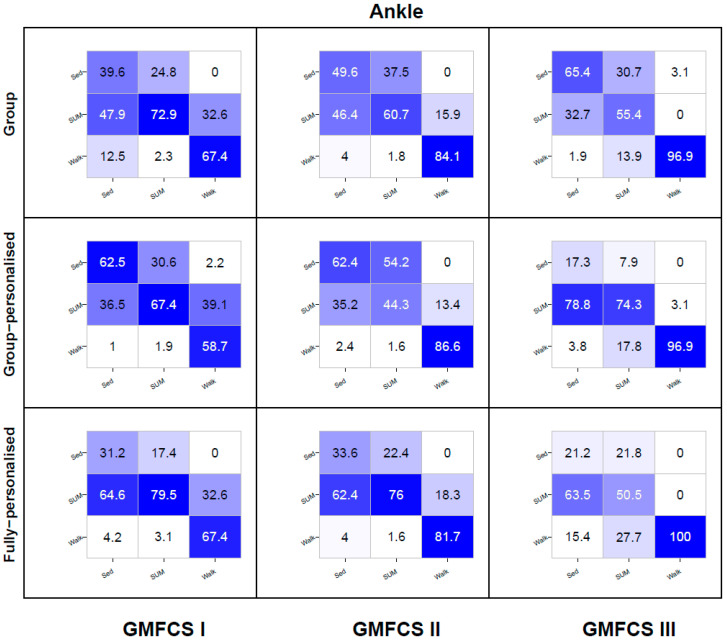
Ankle placement activity class recognition for group, group-personalized, and fully-personalized classification models during the simulated free-living trial. Columns represent observed (%); rows represent predictions (%); values on the diagonal represent correct predictions (%).

**Figure 7:**
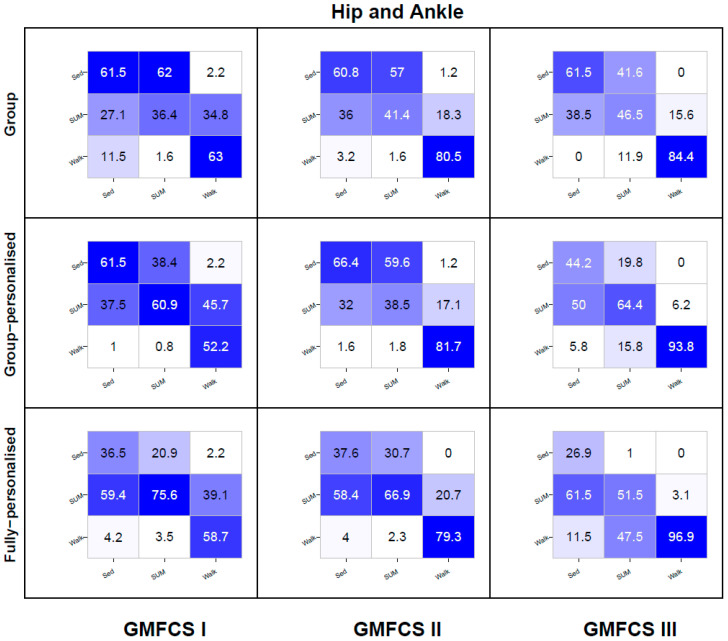
Combined hip and ankle placement activity class recognition for group, group-personalized, and fully-personalized classification models during the simulated free-living trial. Columns represent observed (%); rows represent predictions (%); values on the diagonal represent correct predictions (%).

Heat map confusion matrices by model type and GMFCS level are reported in Figure 5, Figure 6 and Figure 7. To reduce the complexity, only the results for the three best performing accelerometer placement configurations are reported—hip, ankle, and the hip and ankle combined. Detailed confusion matrices for all seven placements by GMFCS level are reported in Supplemental Document 3.

For the hip placement, the recognition of SED was poor to modest for group models (56–60%) at each GMFCS level, modest for group-personalized models (60–65%), and poor for the fully-personalized models (31–49%). For each model type, between 31% to 58% of all SED instances were misclassified as SUM. The recognition of SUM was poor for group models (29–55%) at each GMFCS level, with misclassification frequently occurring as SED (43–69%). Group-personalized models displayed very good recognition of SUM (83.2%) among children at GMFCS III; however, among children classified at GMFCS I and II, recognition was poor (33–40%) and frequently misclassified as SED (59–66%). Fully-personalized models had good to very good recognition (76–84%) and recognition increased as GMFCS function decreased from level I to II. Walking recognition for the group model ranged from modest to good for children at GMFCS levels I and II (65–77%); however, among GMFCS III children, walking recognition was very poor (28%), with 72% of walking instances being misclassified as SUM. Among children at GMFCS II and III, walking recognition for the group-personalized and fully-personalized models ranged from good to excellent (79–97%), with the accuracy increasing as GMFCS function declined from level II to III. Among children at GMFCS I, walking recognition ranged from poor to modest (57–65%) for group-personalized and fully-personalized models.

For the ankle placement, the recognition of SED for the group models was poor among children at GMFCS levels I and II (40–50%), and modest among children at GMFCS III (65%). For group-personalized models recognition was modest among children at GMFCS I and II (62%), but poor among children at GMFCS III (17%). For the fully-personalized models, recognition of SED was poor among all GMFCS levels (21–34%), with just under two-thirds of SED instances being misclassified as SUM. The recognition of SUM was good for group models among children at GMFCS I (73%), but generally poor among children at GMFCS levels II and III (55–61%), with the majority being misclassified as SED (31–38%). Group-personalized models exhibited a good recognition of SUM among children at GMFCS III (74%) and modest recognition among GMFCS I children (67%), but poor recognition among children at GMFCS II (44%). At GMFCS levels I and II, the misclassification of SUM as WALK occurred at rates of less than 2%, but at GMFCS III, this misclassification was 18%. Fully-personalized models exhibited good to very good recognition of SUM among children at GMFCS I and II (76–80%), but poor recognition among children at GMFCS III (51%), with misclassification occurring as either SED (22%) or WALK (28%). For all model types, the recognition of WALK was very good among children at GMFCS II (82–87%) and excellent among children at GMFCS III (97–100%). Among children at GMFCS I, the recognition of WALK was modest (59–67%), with 33–39% of WALK instances being misclassified as SUM.

For the combined hip and ankle (H + A) placement, the recognition of SED by group models was modest (61–62%) at all GMFCS levels. For group-personalized models, the recognition of SED was modest among children at GMFCS I and II (62–66%), but poor among children at GMFCS III (44%). At all GMFCS levels, the recognition of SED for the fully-personalized models was poor (27–38%). For each model type, between 27% and 59% of SED instances were misclassified as SUM. At all GMFCS levels, group models displayed poor recognition of SUM (36–47%), with the majority of misclassifications occurring as SED (42–62%). Group-personalized models exhibited modest recognition of SUM among children at GMFCS levels I and II (61–64%), but poor recognition among children at GMFCS III (39%). At GMFCS levels I and II, the majority of misclassification occurred as SED (38–60%), whilst for children at GMFCS III, misclassification occurred as both SED (20%) and WALK (16%). The recognition of SUM for fully-personalized models was good among GMFCS I children (76%), modest among GMFCS II children (67%), and poor among GMFCS III children (52%), with the majority being misclassified as WALK (48%). For group models, the recognition of WALK was very good among both children at GMFCS II and III (81–84%), but modest among children at GMFCS I (63%). For the group-personalized and fully-personalized models, the recognition of WALK was very good among children at GMFCS II (79–82%) and excellent among children at GMFCS III (94–97%), but poor among children at GMFCS I (52–59%).

## 4. Discussion

To the best of our knowledge, this is the first study to compare group, group-personalized, and fully-personalized activity classification models for children with CP. During cross-validation evaluation, fully-personalized RF activity classification models were more accurate than group and group-personalized models. Fully-personalized models were most accurate among children at GMFCS III, for whom the overall accuracy exceeded 95%, compared to group and group-personalized models, for which the overall accuracy was below 83%. Models trained on ankle data and all two- and three-placement combinations provided a greater overall accuracy greater than those trained on data from the hip or wrist. Irrespective of the model type or placement configuration, however, none of the models performed well when tested under conditions that replicated how activities are performed under real-world conditions.

When evaluated under laboratory conditions, fully-personalized classifiers exhibited a greater accuracy than group and group-personalized classifiers, with the largest performance differential being observed in children at GMFCS III. Furthermore, when children completed activities under simulated free-living conditions, the group-personalized and/or fully-personalized classifiers exhibited a greater accuracy than conventional group classifiers; although, none of the models generalized well and attained a high level of accuracy. These findings are consistent with the notion that the use of personalized classifiers to measure physical activity type and intensity may be advantageous among children with more severe motor impairments, where there is substantial heterogeneity in functional capacity. Notably, more than 90% of children classified at GMFCS III have bilateral CP with spastic, dyskinetic, ataxic, or hypotonic motor impairments and require assistive mobility devices for ambulation [33]. Conversely, most children classified at GMFCS I and II have unilateral CP, with only spastic motor impairment and can ambulate independently. Hence, the common practice of training one-size-fits-all group-based classifiers may not be useful in children with CP.

The fully-personalized, group-personalized, and group RF classifiers trained on laboratory-based activity trials displayed excellent recognition accuracy when cross-validated under laboratory conditions. However, when tested under conditions that replicated real-world conditions, overall accuracy decreased from 89–94% to 5–62%. The substantial decrease in accuracy when tested under simulated free-living conditions is indicative that the cross-validation performance under laboratory conditions is not reflective of a model’s generalizability in real-world scenarios, particularly if the model is trained on data from choreographed activity trials. This supports the findings of prior studies conducted in typically developing children and healthy adults that reported substantial decreases in accuracy when classifiers trained on laboratory-based activity trials were implemented under real-world conditions [27,28,29]. Furthermore, prior studies have demonstrated that when classifiers are trained with free-living data, they have a much better generalizability to real-world conditions [34,35]. Future studies developing machine learning classification models for children with CP should therefore train models using accelerometer data collected under true free-living conditions, where children are allowed to naturally engage in physical activities that are representative of daily activity behaviors. Such studies should have sufficient participants from each GMFCS level to determine if models perform well in children with more severe impairments. Furthermore, to obtain ground-truth activities, video-based direct observation techniques that have been successfully implemented in both the current study and in free-living studies with children should be used to ensure the reliability and precision of activity type labeling [28].

When evaluated under simulated free-living conditions, group-personalized classifiers exhibited greater overall accuracy than the group or fully-personalized classifiers among children at GMFCS III. In contrast, fully-personalized classifiers exhibited greater accuracy than the group and group-personalized models among children at GMFCS levels I and II. The relatively poor performance of the fully-personalized models among children with more severe impairments under simulated free-living conditions was surprising and difficult to explain. When completing the structured activity trials in the laboratory, children functioning at GMFCS III performed sedentary and SUM activities with minimal movement and, subsequently, when the fully-personalized classifier was trained using data from a single individual, there may have been insufficient diversity in the accelerometer data to differentiate these two activity classes. Conversely, group-personalized classifiers trained on data from all GMFCS III participants displayed more diversity in the training data and therefore a greater discriminative ability, which was reflected in the better performance demonstrated during the simulated free-living evaluation. This finding, in combination with the poor generalizability of laboratory-trained classifiers, suggests that fully-personalized classifiers need to be trained on large quantities of accelerometer data collected from an individual completing a wide range of activities under free-living conditions. This, however, significantly increases the research burden placed on both researchers and participants. Alternatively, group-personalized classifiers trained for specific GMFCS classifications can be implemented as an off-the-shelf model; however, such models appear to be less accurate among children with less severe activity limitations.

Random Forest classifiers trained on ankle accelerometer data provided the best overall accuracy during the leave-one-subject-out cross-validation and simulated free-living activity trial. This finding is consistent with prior studies conducted in typically developing children and healthy adults [24,36,37,38]. The distinct rhythmic pattern of walking is easily captured by features from an ankle-worn accelerometer. Consequently, models trained on ankle data had the highest recognition of walking and it was the only placement to able achieve ≥ 95% walking recognition among children at GMFCS III under simulated free-living conditions. Additionally, classification models trained on ankle data provided better recognition of standing utilitarian movements, such as wiping down a counter or playing active video games. For researchers and clinicians interested in monitoring time spent walking, the ankle may be an ideal wear location. An accelerometer can be readily attached to the ankle-foot orthoses commonly worn by this patient group to assist in mobility [39,40]. Prior studies have established that children with CP have a high compliance with wearing ankle-mounted accelerometers, such as the StepWatch, as they do not cause undue irritation or discomfort [41,42,43].

Although the classifiers achieved a high degree of recognition accuracy in the laboratory-based evaluation, SED was frequently misclassified as SUM and vice versa during the simulated free-living trial. During the laboratory-based activity trials, sedentary activities consisted of lying down and quietly sitting and required little movement, while SUM activities such as wiping down a table and playing an active videogame required the children to be standing, while moving side to side or forward and backwards. In contrast, during the simulated free-living trial, children rarely sat quietly during sedentary activities and performed SUM activities with little or no lateral movement. The misclassification of sedentary and SUM during the simulated free-living trial may therefore be attributable, at least in part, to differences in the way that activities were performed in the structured laboratory trials and the simulated free-living evaluation. Regardless of how the activities are performed, a principle difference between the two activity classes is posture. When an accelerometer is placed on a body location that moves into a fixed anatomical plane in different postures, the changes in accelerometer orientation from tilt angle can be used to detect the posture with a high accuracy. Skotte et al. [44] and Edwardson et al. [45] have previously demonstrated that thigh-worn classifiers can differentiate sitting and standing, whereas Gjoreski et al. [46], Tang et al. [47], and Narayanan et al. [48] have shown that a two-placement combination of the thigh and hip/back results in an excellent recognition of lying, sitting, and standing. Prior studies have also demonstrated that thigh-worn monitors, such as activPAL and Uptimer, provide acceptable estimates of sitting and standing among children with CP who have mild motor impairments [49,50]. Although there may be optimal wear locations for a model to recognize certain activities [23], a consideration of the trade-off between activity recognition and wear compliance by children will dictate the optimal wear location for investigators.

The current study had several strengths. To the best of our knowledge, this is the first study to develop and test personalized classifiers in ambulant children with CP that account for significant heterogeneity in relation to movement impairment and functional capacity. Second, the study had sufficient numbers of children classified at GMFCS levels I, II, and III to evaluate classifier performance across the full spectrum of ambulatory ability. Third, classification models were tested under simulated free-living conditions. This allowed for an examination of the models’ performance when activities were completed in sequence, rather than separate activity trials. Opposing these strengths were several limitations. First, although the classifiers were evaluated under simulated free-living conditions that were intended to replicate a real-world scenario, the tasks performed and brief duration did not fully replicate the activity performances of children with CP in true free-living contexts. Consequently, future studies should evaluate the performance of group and personalized classifiers under true free-living conditions. Second, the study did not include a thigh placement. The propensity for classifiers trained on hip and wrist accelerometer data to misclassify sitting and standing is well-documented [51,52,53] and the inclusion of posture-related features from a thigh-mounted accelerometer may have improved the recognition of SED and SUM in the simulated free-living evaluation trial. Third, the current study only trained RF classifiers and did not benchmark the performance with other supervised or unsupervised learning algorithms. RF classifiers were chosen in the current study because they are ensemble learning models which have been shown to provide accurate activity recognition in children with CP [22,54], as well as those with typical development [55,56]. As the aim of the current study was to evaluate the influence of personalization on the classification accuracy, it was important that models were trained using the same supervised learning algorithm. Future studies could examine the performance of other machine learning algorithms and benchmark the performance to the results observed in this study. The final group and group-personalized models with the annotated dataset and code for implementation are available at https://github.com/QUTcparg/Sensors_CP_PersonalisedModels.

## 5. Conclusions

In summary, when evaluated under laboratory conditions, group-personalized and fully-personalized RF activity classification models provide a more accurate recognition of physical activity in children with CP than “one-size-fits-all” group models. Personalized models yielded the greatest improvement in accuracy among children with the more severe motor impairments. When evaluated under simulated free-living conditions, personalized models exhibited a higher classification accuracy than conventional group models; however, the performance for all models declined substantially. Accordingly, future studies should evaluate the feasibility and comparative accuracy of group-personalized and fully-personalized activity classification models trained on accelerometer data collected under true free-living conditions.

## Figures and Tables

**Table 1:** Participant characteristics.

	GMFCS I (N = 10)	GMFCS II (N = 20)	GMFCS III (N = 8)
Sex			
Male N (%)	4 (40%)	7 (35%)	2 (25%)
Female N (%)	6 (60%)	13 (65%)	6 (75%)
Motor Distribution			
Hemiplegia N (%)	8 (80%)	13 (65%)	0 (0%)
Diplegia N (%)	2 (20%)	7 (35%)	5 (62.5%)
Quadriplegia N (%)	0 (0%)	0 (0%)	3 (37.5%)
Age (y)	10.8 ± 1.8	12.0 ± 3.1	11.8 ± 4.5
Height (cm)	145.1 ± 9.9	147.2 ± 16.7	129.7 ± 19.2
Weight (kg)	40.8 ± 12.6	42.3 ± 15.6	34.9 ± 17.8

GMFCS = Gross Motor Function Classification System.

**Table 2:** LOSO-CV overall and activity class accuracy for group, GMFCS-specific, and personalized classification models.

		GMFCS I						GMFCS II						GMFCS III					
		G		GP		FP		G		GP		FP		G		GP		FP	
		Mean (SD)		Mean (SD)		Mean (SD)		Mean (SD)		Mean (SD)		Mean (SD)		Mean (SD)		Mean (SD)		Mean (SD)	
Wrist	SED	92.7	(13.5)	94.1	(15.0)	99.3	(0.4)	94.7	(9.5)	93.2	(12.2)	99.0	(0.3)	85.0	(27.6)	57.7	(38.4)	99.1	(1.2)
	SUM	89.3	(10.0)	87.0	(9.5)	97.0	(2.3)	82.6	(14.6)	79.6	(20.1)	96.2	(1.2)	76.7	(15.0)	71.4	(25.5)	96.3	(1.1)
	WALK	96.2	(6.6)	91.9	(12.4)	98.4	(0.9)	90.4	(16.4)	92.6	(13.7)	97.2	(0.6)	84.2	(14.1)	75.5	(32.4)	96.2	(1.2)
Hip	SED	94.4	(12.5)	89.1	(16.0)	100.0	(0.1)	92.1	(13.2)	94.4	(10.2)	100.0	(0.3)	89.3	(22.7)	76.9	(34.2)	100.0	(0.4)
	SUM	83.8	(22.2)	76.8	(28.7)	98.5	(0.8)	87.6	(18.6)	85.3	(21.8)	98.4	(0.4)	75.1	(21.3)	73.3	(17.6)	98.2	(0.2)
	WALK	99.6	(1.1)	88.3	(11.2)	99.2	(0.3)	97.3	(3.9)	94.4	(10.1)	98.8	(0.9)	53.5	(34.7)	65.5	(38.5)	98.9	(0.2)
Ankle	SED	95.8	(5.3)	97.6	(2.9)	100.0	(0.1)	94.7	(12.9)	96.6	(11.6)	100.0	(0.3)	89.8	(26.9)	78.5	(37.8)	100.0	(0.1)
	SUM	91.9	(16.2)	91.1	(10.0)	98.8	(1.3)	90.4	(15.3)	93.2	(7.9)	99.2	(0.6)	71.6	(23.1)	74.5	(23.6)	99.1	(0.8)
	WALK	99.6	(1.1)	99.0	(2.3)	98.7	(1.5)	98.0	(2.4)	96.8	(5.5)	99.4	(0.5)	84.0	(16.8)	96.3	(4.4)	99.0	(1.1)
W + H	SED	89.2	(22.6)	94.0	(14.0)	100.0	(0.2)	94.4	(11.9)	94.9	(11.2)	100.0	(0.2)	99.6	(1.1)	75.0	(30.2)	100.0	(0.4)
	SUM	90.8	(16.9)	87.0	(25.4)	99.1	(1.3)	85.2	(17.6)	88.1	(20.2)	99.3	(1.2)	92.1	(10.4)	80.4	(15.2)	99.8	(0.7)
	WALK	90.9	(14.9)	98.3	(2.5)	99.3	(1.1)	94.9	(11.9)	96.2	(5.3)	99.1	(1.3)	95.8	(4.4)	94.1	(8.1)	99.7	(0.4)
W + A	SED	94.2	(12.2)	98.4	(3.2)	100.0	(0.2)	93.9	(12.1)	97.3	(10.0)	100.0	(0.1)	92.0	(18.6)	80.1	(33.4)	100.0	(0.2)
	SUM	91.6	(11.4)	91.9	(12.3)	98.1	(2.3)	93.8	(10.4)	93.7	(6.5)	99.2	(1.7)	68.6	(23.2)	81.1	(14.9)	99.5	(1.0)
	WALK	99.6	(1.1)	99.0	(2.3)	98.4	(1.4)	97.6	(3.7)	97.9	(2.8)	99.4	(1.9)	90.5	(9.9)	96.8	(3.7)	98.2	(1.7)
H + A	SED	94.6	(10.5)	96.9	(7.0)	100.0	(0.1)	96.3	(7.9)	96.5	(11.7)	100.0	(0.2)	86.9	(27.1)	76.3	(35.2)	100.0	(0.2)
	SUM	87.3	(21.7)	89.4	(14.1)	98.6	(1.6)	93.8	(12.9)	92.1	(11.1)	98.2	(1.3)	70.5	(20.6)	73.6	(24.1)	99.4	(0.7)
	WALK	99.6	(1.1)	98.6	(3.3)	99.3	(0.8)	98.3	(2.3)	97.4	(2.4)	98.9	(1.4)	87.8	(13.6)	96.3	(4.5)	99.2	(0.4)
W + H + A	SED	95.3	(10.4)	96.4	(7.9)	100.0	(0.2)	96.2	(9.6)	97.0	(7.2)	100.0	(0.2)	87.5	(27.6)	81.3	(32.7)	100.0	(0.2)
	SUM	91.9	(12.9)	91.0	(12.4)	99.3	(1.2)	93.8	(8.5)	91.4	(10.7)	99.1	(0.7)	77.2	(19.8)	82.1	(14.7)	98.8	(1.4)
	WALK	99.6	(1.1)	98.8	(2.8)	99.5	(0.4)	98.1	(2.8)	98.2	(2.6)	99.2	(0.9)	89.4	(11.1)	96.8	(3.7)	99.0	(1.1)

G = group; GP = group-personalized; GF = fully-personalized; W + H = wrist and hip; W + A = wrist and ankle; H + A = hip and ankle; W + H + A = wrist, hip, and ankle; SED = sedentary; SUM = standing utilitarian movement; WALK = walking.

## Supplementary Materials

The following are available online at https://www.mdpi.com/1424-8220/20/14/3976/s1.
