# Supplementary material for: Machine Learning to Quantify Physical Activity in Children with Cerebral Palsy: Comparison of Group, Group-Personalized, and Fully-Personalized Activity Classification Models
Source: Sensors (Basel). 2020 Jul 17;20(14):3976. doi: 10.3390/s20143976 (PMC7411900; doi:10.3390/s20143976)
Supplement: Supplementary file 1 [file sensors-20-03976-s001.pdf]

Supplemental Document 1: Features selected for each placement and combination of placements

Feature	Wrist	Hip	Ankle	W+H	W+A	H+A	W+H+A
Min			Z			Y(a)	X(a)
Max	Y				X(a)	X(h)	Y(a), X(a)
Mean	Y						
Var	X	Y, X, Z	X, Y, Z	Z(w), Z(w)			
SD	Y, Z	Y, X, Z	X, Y, Z	Y(h), X(h), Z(h)	Z(a), X(a)	Z(h), X(h)	X(h), Y(a), Z(a), X(a)
Skewness							
Kurtosis							
25th percentile	Y						
50th percentile	Y						
75th percentile	Y			Y(w)			
Zero-crossings	Y	Z		X(h), Z(h), Y(w)	Z(a)	Z(h)	Z(h), Y(h), Z(a)
Energy	Y			Y(h), Z(w), Y(w)	Z(w), Y(a), Y(w)	Y(h)	Y(h), Y(a), Z(w), Y(w)
Dom. Fr	Y	X, Z, Y					
Dom. Mag	Y	Y, X					
Entropy	Y, Z	Y, X, Z	Y, X, Z	X(h), Y(h), Y(w)	X(a), Y(w), Y(a)	X(a), X(h), Y(a), Y(h)	Z(h), Z(a), X(h), X(a), Y(a), Y(h)
XY corr							
XZ corr							
YZ corr							
Mean VM	✓						

Y = y-axis; X = x-axis; Z = z-axis; VM = vector magnitude; w= wrist; h= hip; a = ankle; min = minimum; max = maximum; var = variance; SD = standard deviation; Dom. Fr = dominant frequency; Dom. Mag. = dominant magnitude; VM = vector magnitude
